# FGF-2 induces the proliferation of human periodontal ligament cells and modulates their osteoblastic phenotype by affecting Runx2 expression in the presence and absence of osteogenic inducers

**DOI:** 10.3892/ijmm.2015.2271

**Published:** 2015-07-01

**Authors:** SHAOFENG AN, XIANGYA HUANG, YAN GAO, JUNQI LING, YIHUA HUANG, YIN XIAO

**Affiliations:** 1 Department of Operative Dentistry and Endodontics, Guanghua School of Stomatology, Hospital of Stomatology, Sun Yat-sen University and Guangdong Provincial Key Laboratory of Stomatology, Guangzhou, Guangdong 510055, P.R. China; 2 Institute of Health and Biomedical Innovation, Queensland University of Technology, Brisbane, QLD 4059, Australia

**Keywords:** basic fibroblast growth factor-2, human periodontal ligament cells, osteogenic inducers, osteogenic differentiation, epithelial differentiation

## Abstract

The exact phenotype of human periodontal ligament cells (hPDLCs) remains a controversial area. Basic fibroblast growth factor (FGF-2) exhibits various functions and its effect on hPDLCs is also controversial. Therefore, the present study examined the effect of FGF-2 on the growth and osteoblastic phenotype of hPDLCs with or without osteogenic inducers (dexamethasone and β-glycerophosphate). FGF-2 was added to defined growth culture medium and osteogenic inductive culture medium. Cell proliferation, osteogenic differentiation and mineralization were measured. The selected differentiation markers, *Runx2*, collagen type I, α1 (*Col1a1*), osteocalcin (OCN) and epidermal growth factor receptor (*EGFR*), were investigated by reverse transcription-quantitative polymerase chain reaction (RT-qPCR). Runx2 and OCN protein expression was measured by western blotting. FGF-2 significantly increased the proliferation of hPDLCs, but did not affect alkaline phosphatase activity. RT-qPCR analysis revealed enhanced mRNA expression of *Runx2*, OCN and *EGFR*, but suppressed *Col1a1* gene expression in the absence of osteogenic inducers, whereas all these gene levels had no clear trend in their presence. The Runx2 protein expression was clearly increased, but the OCN protein level showed no evident trend. The mineralization assay demonstrated that FGF-2 inhibited mineralized matrix deposition with osteogenic inducers. These results suggested that FGF-2 induces the growth of immature hPDLCs, which is a competitive inhibitor of epithelial downgrowth, and suppresses their differentiation into mineralized tissue by affecting Runx2 expression. Therefore, this may lead to the acceleration of periodontal regeneration.

## Introduction

Periodontitis is a highly prevalent inflammatory oral disease. Delayed treatment for aggressive periodontitis could result in the loss of soft connective tissues and bone that surrounds the teeth and is a major cause of tooth loss in adults (1). The ideal aim of periodontal therapy is achieving simultaneous regeneration of the lost alveolar bone, cementum and periodontal soft tissues (2). Current treatment options, such as professional tooth cleaning/scaling/polishing, root planning, periodontal debridement and guided tissue regeneration, used either alone or in combination, only have a limited success in impeding periodontitis, particularly at the advanced stage of the disease (2–4). Together with the recent advances in periodontal tissue engineering, novel treatment methods utilizing signaling molecules and/or cell-based therapies have been developed as new strategies to regenerate periodontal tissue damaged by periodontitis (2,5,6).

Human periodontal ligament cells (hPDLCs) are believed to be key players during periodontal tissue regeneration. Numerous studies have shown that hPDLCs can be committed to several cell lineages, including osteoblastic, fibroblastic, and cementoblastic (7–10). In addition, the aforementioned studies indicate that the organic osteogenic inductive factors, dexamethasone (DEX) and β-glycerophosphate (β-GP), are efficient for stimulating human osteoblasts (HOBs) and mesenchymal stem cells (MSCs) *in vitro*, but are not believed to be able to achieve similar stimulation *in vivo*. Additionally, the exact phenotype of hPDLCs remains a topic of controversy, in particular, concerning whether these cells are similar to a terminally differentiated cell type with a fibroblastic nature or a progenitor cell that potentially can be induced into a fibroblastic or osteoblastic phenotype by certain growth factors (11).

Basic fibroblast growth factor (FGF-2) is reported to be a strong mitogen and acts on various cell types, including HOBs, MSCs and hPDLCs, in addition to being effective in accelerating the proliferation of fibroblasts and osteoblasts, and enhancing angiogenesis. These biological actions are directly associated with periodontal tissue regeneration (12–14).

The optimum aims of periodontal treatment are to regenerate the destroyed cementum, alveolar bone and periodontal ligament, while also preventing epithelization during wound healing (2,15,16). Numerous studies have demonstrated that the topical application of FGF-2 promotes the healing of destructive periodontal tissue without ankylosis, root resorption or epithelial downgrowth in experimental alveolar bone defects in the beagle dog and primate models (16–22). However, FGF-2 is considered a multifunctional growth factor that has diversified actions and its effect in the differentiation of hPDLCs remains controversial (12). hPDLCs are a heterogeneous population of cells and the exact function of FGF-2 in stabilizing the fibroblastic phenotype and maintaining the human periodontal ligament (hPDL) structural integrity remains largely unknown. Based on the above factors and our previous study (23), we hypothesize that in the present experimental system (with or without osteogenic inducers), FGF-2 may be able to maintain the fibroblastic phenotype of hPDLCs and prevent them from differentiating into mature osteoblasts.

Thus, the aim of the present study was to reveal the molecular and cellular mechanisms by which FGF-2 affects the osteoblastic and fibroblastic phenotypes of hPDLCs in the presence and absence of osteogenic inducers. The knowledge gained from this study may facilitate the development of purposeful strategies to influence hPDLC reparative capacities.

## Materials and methods

### Cell culture

hPDLCs were isolated from healthy premolar teeth using a previously described method (23). Healthy subjects <20 years old undergoing orthodontic treatment were recruited with the understanding and consent for the isolation of hPDLCs. All the experimental protocols used were approved by the Ethics Committee of Sun Yat-sen University (Guangdong, China). Briefly, fresh PDL tissues were collected rapidly following the tooth extraction. Under sterile conditions, the PDL tissue was washed with sterile phosphate-buffered saline (PBS) and scraped from the middle-third of the root with a scalpel. The tissue was subsequently chopped into smaller pieces and soaked in fresh Dulbecco's modified Eagle's medium [DMEM/high glucose; Hyclone Biochemical Product (Beijing) Co., Ltd., Beijing, China] containing 20% fetal bovine serum (FBS) (Biological Industries Israel Beit-Haemek, Ltd., Kibbutz Beit-Haemek, Israel) and 2% (v/v) penicillin/streptomycin (Invitrogen Life Technologies, Carlsbad, CA, USA). An explant culture method was used in the primary culture. The medium was changed every 3 days. Cells typically emerged from the tissues within 1–2 weeks of being explanted in a humidified atmosphere of 5% CO_2_ at 37°C. When the cells growing from the explants reached ~80% confluency, they were subcultured at a 1:3 ratio through trypsinization (trypsin/EDTA; Invitrogen Life Technologies). Only the fifth passage of hPDLCs was used in subsequent experiments.

### FGF-2 treatment

Recombinant Human FGF-basic (FGF-2) (Peprotech, Inc., Rocky Hill, NJ, USA) was added to the defined growth culture medium and osteogenic inductive culture medium. There were four treatment groups: i) hPDLCs + growth medium (GM) (DMEM containing 10% FBS and 2% penicillin/streptomycin); ii) hPDLCs + GM + 20 ng/ml FGF-2 (GM + FGF-2); iii) hPDLCs + osteogenic medium (OM) [DMEM with 10% FBS, 2% (v/v) penicillin/streptomycin, 10^−8^ M DEX, 50 *µ*g/ml ascorbic acid and 8 mM β-GP]; and iv) hPDLCs + OM + 20 ng/ml FGF-2 (OM + FGF-2).

### Cell proliferation assay

hPDLC viability was monitored using a Cell Counting kit-8 (CCK-8) (Beyotime, Shanghai, China). Briefly, hPDLCs at passage 5 were seeded in 96-well plates at an initial density of 3×10^3^ cells/well in medium, with five replicates for each group. The medium was replaced every 3 days. After 1, 2, 3, 5 and 7 days of subculture in medium treated differently, 20 *µ*l of CCK-8 solution was added to every well and incubated with the cells for 4 h in a humidified atmosphere of 5% CO_2_ at 37°C. The absorbance of each sample was determined at the wavelength of 450 nm on a 96-well plate reader.

### Alkaline phosphatase (ALP) activity assay

hPDLCs were seeded in 24-well plates in triplicate at a density of 2×10^4^ cells/well and cultured in defined media subsequent to reaching ~80% confluency. Following 3, 7, 14 and 21 days in culture, an ALP activity assay kit (Nanjing Jiancheng Biotechnology Co., Ltd., Jiangsu, China) was used to detect ALP activity of hPDLCs, following the manufacturer's instructions. In brief, the cell layers in plates were washed three times with 10 mM sterile PBS, and subsequently a small amount (1 ml) of cold 10 mM Tris-HCl buffer (pH 7.4) containing 0.1% Triton X-100 was added to the culture well, and the mixture was lysed through two freeze (−20°C)/thaw cycles. The supernatant (50 *µ*l/well) was placed into 24-well plates for the ALP activity assay using 50 *µ*l of an ALP substrate solution (16 mM *p*-nitrophenyl phosphate and 2 mM MgCl_2_). After a 30-min incubation at 37°C, the reaction was stopped by the addition of 50 *µ*l of 0.2 M NaOH, and the absorbance of the reaction product (*p*-nitrophenol) was read at 520 nm.

### Reverse transcription-quantitative polymerase chain reaction (RT-qPCR) analysis

After 7 and 14 days of culture, the expression levels of selected osteoblastic and fibroblastic genes were determined in triplicate by RT-qPCR. Total RNA was extracted from hPDLCs using TRIzol Reagent (Invitrogen Life Technologies) and was quantified in a spectrophotometer by absorbance readings at 260 nm. The extracted total RNA (2 *µ*g) was treated with 1 unit DNase I for cDNA synthesis in each RT-qPCR using the RevertAid™ First Strand cDNA Synthesis kit (K1622; MBI Fermentas, Inc., Burlington, ON, Canada). PCR was carried out using a combination of 2.5 *µ*l of diluted cDNA and 12.5 *µ*l of SYBR-Green Real-time PCR Master mix (QPK-201; Toyobo Co., Ltd., Osaka, Japan) in a Chromo4 System (Bio-Rad, Hercules, CA, USA). The sequential reaction conditions were as follows: 95°C for 1 min denaturation followed by 40 cycles of denaturation at 95°C for 15 sec, annealing at 60°C for 15 sec and extension at 72°C for 30 sec. The specificity of the PCR products was confirmed by melting curve analysis (60–93°C, read every 0.4°C, hold 1 sec). GAPDH was used as the reference gene for data normalization. The number of cDNA copies was calculated with the apparent cycle threshold (CT). The ΔCT value expresses the difference between the CT of the target gene and the CT of GAPDH from the same sample: ΔCT = the target gene CT - GAPDH CT, which can be expressed as a percentage of GAPDH. The relative expression level of the target gene (fold of the reference gene) was obtained by transforming the logarithmic values to absolute values using 2^−ΔCT^. The primer sequences are shown in Table I.

### Western blotting

After 7 and 14 days of culture, Runx2 and osteocalcin (OCN) protein expression was measured using western blotting. In brief, hPDLCs were washed three times with pre-cooling PBS. The constructs were subsequently homogenized in sodium dodecyl sulfate polyacrylamide gel electrophoresis (SDS-PAGE) sample buffer, and cellular protein was extracted in lysis buffer [50 mM Tris-HCl, 0.5% Triton X-100, 2 mM EDTA and 150 mM NaCl (pH 7.5)] containing phenylmethylsulfonyl fluoride. The protein samples (30 *µ*g) were electrophoresed through 10% SDS-PAGE and transferred to a polyvinylidene fluoride (PVDF) membrane (Millipore, Bedford, MA, USA) in a wet blotting apparatus for 1.5 h at 300 mA. The PVDF membrane was subsequently blocked with 5% bovine serum albumin (Wuhan Boster Biological Technology, Ltd., Hubei, China) for 1 h at room temperature, and the blot was incubated with 1:1,000 dilution of anti-human Runx2 antibody (Cat. no. AF2006; R&D Systems, Minneapolis, MN, USA) and 1:500 of anti-human OCN (Cat. no. ab10911; Millipore, Billerica, MA, USA) for 2 h, washed with Tris-buffered saline Tween 20 (TBST) four times and incubated with a 1:2,500 dilution of horseradish peroxidase (HRP) AffiniPure goat anti-rabbit secondary antibody (Santa Cruz Biotechnology, Inc., Dallas, TX, USA) for 1 h, and further washed four times with TBST. HRP detection was accomplished via chemical luminescence exposed to X-ray film with an ECL western blotting detection system.

### Calcium content quantitative assay

The calcium content of the cells was measured by a colorimetric quantitative method using a Calcium Assay kit (Shanghai Genmed Gene Pharmaceutical Technology Co., Ltd., Shanghai, China) following the manufacturer's instructions. Cells were seeded at a cell density of 5×10^4^ cells/well in 12-well tissue culture plates. After 14 and 21 days of culture, deposition of calcium in the cell layer was performed in triplicate. The absorbances of dyes were read at a wavelength of 595 nm using a UV/Visible light Spectrophotometer (Varian Cary 50; Varian Australia Pty, Ltd., Mulgrave, VIC, Australia).

### Alizarin Red S staining

After 7, 14 and 21 days of culture, the mineralized matrix nodules were detected using an Alizarin Red S Staining kit (Shanghai Genmed Gene Pharmaceutical Technology Co., Ltd.) following the manufacturer's instructions. Prior to staining, the cell cultures were washed five times with 10 mM sterile PBS and fixed using 10% (v/v) neutral buffered formalin for 30 min. Subsequently, the samples were stained for 5 min and the excess stain was rinsed with PBS. An Inverted Phase Contrast Microscope (Olympus IX41; Olympus Corp., Tokyo, Japan) was used to observe the stained cells.

### Statistical analysis

All the values are reported as the mean ± standard deviation. Statistical analysis of the data was performed using the SPSS 17.0 software package (SPSS, Inc., Chicago, IL, USA). One-way analysis of variance and the least significant difference test were used for parameter estimation and hypothesis testing. P<0.05 was considered to indicate a statistically significant difference.

## Results

### FGF-2 promotes the proliferation of hPDLCs

**Figure 1:**
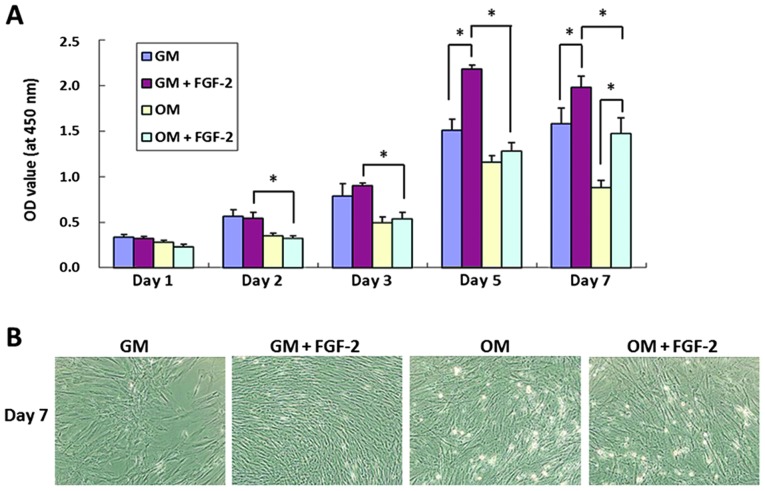
Cell proliferation of human periodontal ligament cells (hPDLCs) cultured in defined media. (A) At days 1 and 2, basic fibroblast growth factor (FGF-2) suppressed the cell proliferation rate. From day 3 onwards, hPDLCs cultured in the growth medium (GM) with added FGF-2 exhibited maximal levels of proliferation, and those in the osteogenic medium (OM) exhibited minimum levels. Supplementing media with FGF-2 enhanced cell proliferation in the absence and presence of osteogenic inducers. ^*^P<0.05 against the control. (B) Representative images of the growth of hPDLCs on day 7. The GM + FGF-2 group had the largest number of cells. Mineralization nodes (the white dots) appeared in the OM and OM + FGF-2 groups (original magnification, ×200).

At days 1 and 2, FGF-2 had no significant effect on the cell proliferation rate (P>0.05). From day 3 onwards, hPDLCs cultured in the GM with added FGF-2 exhibited the maximal levels of proliferation, and those in the OM exhibited minimum levels (P<0.05) (Fig. 1). This suggested that supplementing growth media with FGF-2 enhanced proliferation of hPDLCs in the presence and absence of osteogenic inducers.

### FGF-2 does not affect ALP activity

**Figure 2:**
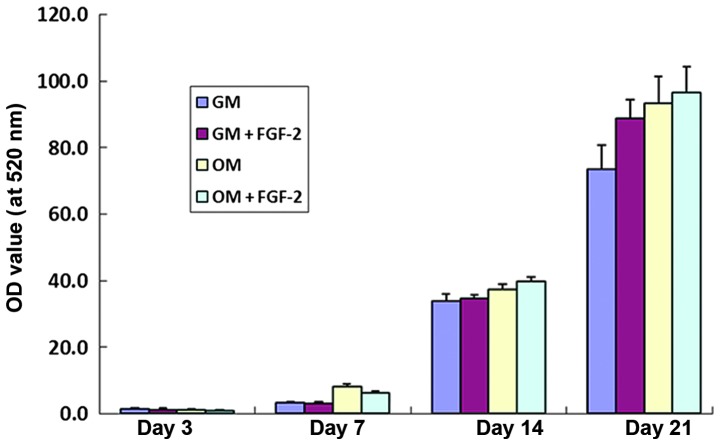
Alkaline phosphatase (ALP) activity of human periodontal ligament cells (hPDLCs) cultured in defined media. The ALP activity progressively increased with culture time in all the groups. At days 3 and 7, basic fibroblast growth factor (FGF-2) inhibited the ALP activity in the absence [growth medium (GM)] and presence of osteogenic inducers [osteogenic medium (OM)]. However, FGF-2 promoted ALP activity at days 14 and 21, however, there were no significant differences at any time-points.

The specific ALP activity progressively increased with the duration of culture time in all the groups. At days 3 and 7 of culture, FGF-2 inhibited the ALP activity. However, FGF-2 promoted the ALP activity at days 14 and 21. There were, however, no significant differences between FGF-2-treated and -untreated groups at any time-points (P>0.05) (Fig. 2).

### FGF-2 affects the mRNA and protein expression of Runx2, but not the OCN protein level

**Figure 3:**
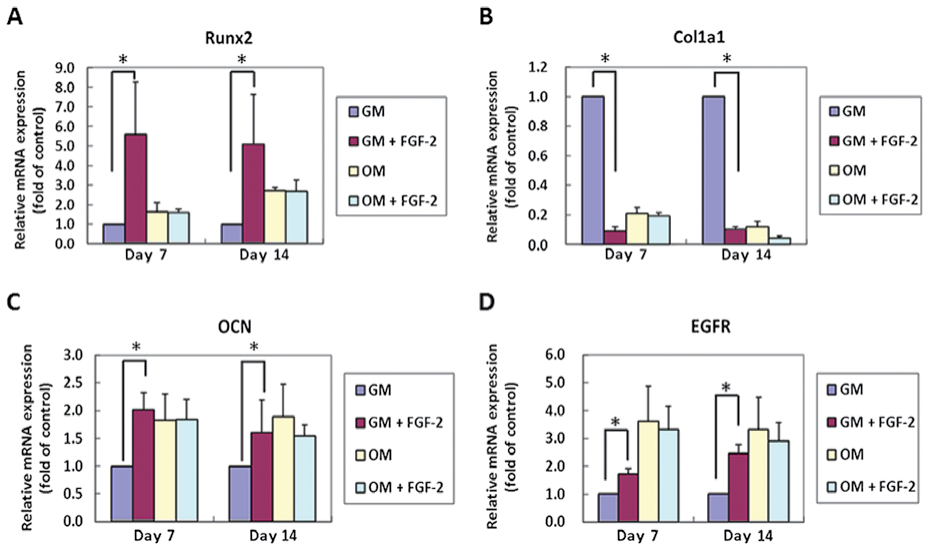
Reverse transcription-quantitative polymerase chain reaction (RT-qPCR) analysis of osteogenic and fibroblastic differentiation markers in human periodontal ligament cells (hPDLCs). The results are standardized to the reference gene *GAPDH*, and expressed as relative mRNA levels. (A) *Runx2* mRNA. In the cultures lacking osteogenic inducers [growth medium (GM)], the *Runx2* mRNA expression was upregulated significantly at days 7 and 14. However, in the osteogenic medium (OM), there were no clear trends in the levels of *Runx2*. (B) Collagen type I, α1 (*Col1a1*) mRNA. In the GM, that is, in the absence of osteogenic inducers, basic fibroblast growth factor (FGF-2) suppressed *Col1a1* mRNA levels; however, no no significant effect was shown in the OM (in the presence of osteogenic inducers). (C) Osteocalcin (OCN) mRNA. In the GM, lacking osteogenic inducers, FGF-2 upregulated the gene expression of OCN significantly at day 7. However, in the OM supplemented with osteogenic inducers, there were no clear trends. (D) Epidermal growth factor receptor (*EGFR*) mRNA. FGF-2 promoted the expression of the *EGFR* gene in the absence of osteogenic inducers; however, there was inhibited *EGFR* gene expression in the presence of osteogenic inducers.

In the cultures without osteogenic inducers, *Runx2* mRNA expression was significantly upregulated under the stimulation of FGF-2 at days 7 and 14 (P<0.05). However, in the OM, there was no evident effect on *Runx2* expression (P>0.05) (Fig. 3A).

**Figure 4:**
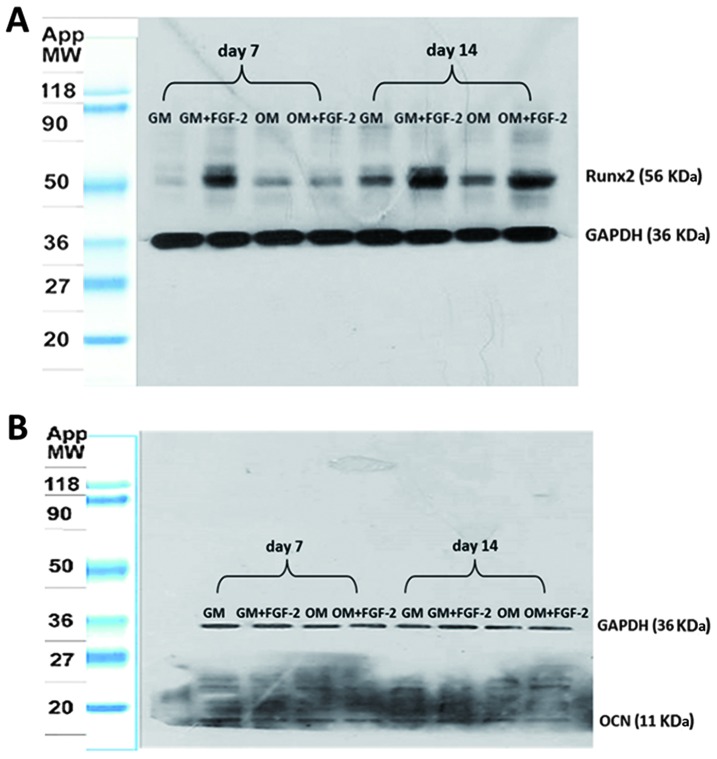
Western blotting of Runx2 and osteocalcin (OCN) expression in human periodontal ligament cells (hPDLCs). (A) In the western blotting assay, basic fibroblast growth factor (FGF-2) enhanced the Runx2 expression at days 7 and 14 in the growth medium (GM). In the osteogenic medium (OM), the Runx2 protein level exhibited no clear changes at day 7; however, there was a significant enhancement at day 14. (B) The protein level of OCN showed no evident change at days 7 and 14 in the GM and the OM.

In the western blot analysis, Runx2 expression was increased at days 7 and 14 in the medium without osteogenic inducers under the stimulation of FGF-2. In the OM samples, there was no increase in Runx2 protein levels at day 7, whereas at day 14 there was a significantly increased protein expression stimulated by FGF-2 (Fig. 4A). However, the protein expression of OCN showed no evident changes at day 7 and 14 (Fig. 4B).

### FGF-2 affects collagen type I, α1 (Col1a1), OCN and epidermal growth factor receptor (EGFR) gene expression of hPDLCs in the absence of osteogenic inducers

There was a significant decrease in *Col1a1* mRNA levels in the GM + FGF-2 samples (P<0.05), whereas in the OM samples the gene expression was downregulated at day 7 (P>0.05) under the stimulation of FGF-2 (Fig. 3B). OCN and *EGFR* expression was upregulated in GM + FGF-2 (P<0.05); but downregulated to various degrees in the OM + FGF-2 samples (P>0.05) (Fig. 3C and D). Compared with the GM condition, OM culture stimulated OCN and *EGFR* expression in the absence of FGF-2.

### FGF-2 suppresses the mineralization of hPDLCs in the presence of osteogenic inducers

**Figure 5:**
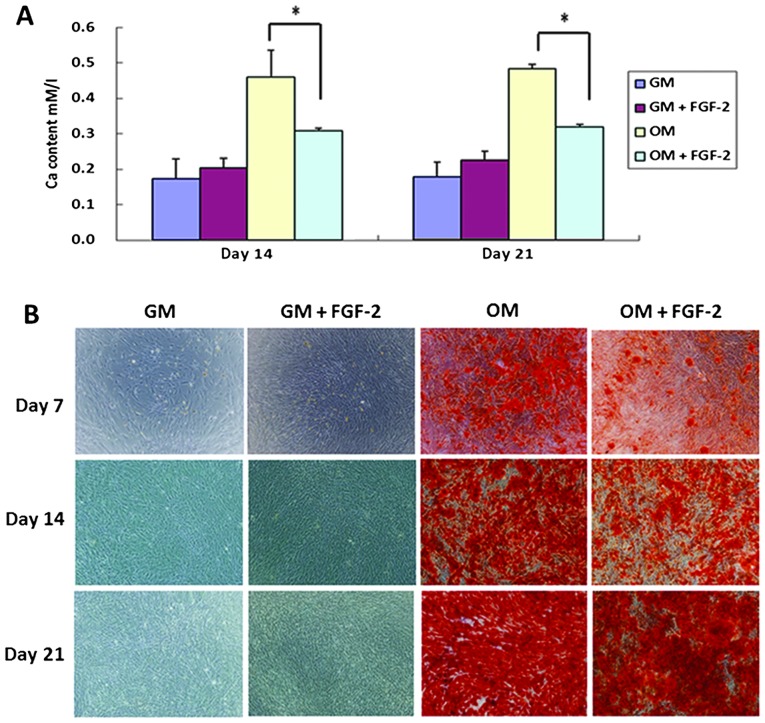
Calcium content and mineralization of human periodontal ligament cells (hPDLCs) in the media with different supplements. (A) Calcium content assay. The calcium content in the experimental groups treated with basic fibroblast growth factor (FGF-2) was higher than that in the untreated growth medium (GM). However, in the osteogenic medium (OM), FGF-2 significantly suppressed the calcium content. ^*^P<0.05 against the control. (B) Alizarin Red S staining. Mineralized nodes were observed only in the OM. FGF-2 inhibited calcium deposition at days 7, 14 and 21. Images are representative of the groups at days 7, 14 and 21 (original magnification, ×100).

After 14 and 21 days of culture, the calcium content in the cultures was measured. The calcium content in the FGF-2-treated groups was higher than the non-FGF-2 groups (P>0.05). However, in the OM + FGF-2 group there was a significant reduction of the calcium content (P<0.05) (Fig. 5A).

Alizarin Red S staining indicated that mineralized nodes were observed only in the OM, and mineralized matrix deposition gradually increased with time in culture. The addition of FGF-2 resulted in reduced calcium deposition as evaluated at days 7, 14 and 21 (Fig. 5B).

## Discussion

Cell proliferation and differentiation are two crucial aspects of cell-based bone regeneration. Osteogenic-differentiated cells generally exhibit low proliferation rates (24) and may explain why hPDLCs cultured in medium with added osteogenic inducers exhibited the lowest proliferation rate. hPDLCs in groups without osteogenic inducers had higher *EGFR* mRNA levels, which resulted in increased cell proliferation most possibly via activation of its downstream targets, ERK1/2 and Akt (25,26). Thus, the cell proliferation rates in this group are higher compared to the OM group. CCK-8 analysis indica ted that FGF-2 was able to increase the proliferation rate of hPDLCs in the presence and absence of osteogenic inducers. These results are similar to those of a study that identified that co-stimulation with fetal calf serum synergistically enhanced FGF-2-induced periodontal ligament cell proliferation, but inhibited FGF-2-induced proliferation of gingival epithelial cells (27). We speculate that this enhancement could provide an alternate route to limit the downgrowth of junctional epithelium and increase the relatively low number of cells adhering to the scaffold surface in periodontal tissue engineering.

ALP activity was a well-defined marker for osteogenic differentiation. Previous studies indicate that the addition of FGF-2 significantly decreased ALP activity and calcified nodule formation in hPDLCs in a dose-dependent manner (12). The concentration of FGF-2 chosen in the present study at 20 ng/ml was based on the previous studies (28,29). In these studies, it was reported that FGF-2 promoted the proliferation of hPDLCs in a dose-dependent manner and that the action plateaued c at >100 ng/ml, whereas ALP activities of hPDLCs were completely prohibited when the cells were treated with >10 ng/ml of FGF-2. The present data indicated that FGF-2 inhibited calcified nodule formation; however, it did not affect the ALP activity of hPDLCs. This difference may be associated with the concentration of FGF-2 and the experimental conditions.

Endogenous *Runx2* is expressed in pre-osteoblasts, immature osteoblasts, early mature osteoblasts and pre-odontoblasts, and has been served as a master regulator in osteoblastic differentiation and bone formation (30). The present results showed that there was no observable effect of FGF-2 on *Runx2* gene expression regardless of FGF-2-induced Runx2 protein expression on day 14. We hypothesize that this phenomenon may be explained by the study of Shui *et al* (31), which reported that osteoblastic differentiation of human mesenchymal stem cells (hMSCs) is associated primarily with increases of Runx2 activity through a post-translational mechanism without a change in mRNA.

Col1a1 is the most abundant organic component of dentin, hPDL and bone, and is an essential factor in the formation of calcified nodules (32). The overexpression of Runx2 inhibits osteoblast terminal differentiation and alters their expression of extracellular matrix protein genes, which results in down-regulation of *Col1a1* mRNA (30,33). In the present study, FGF-2 inhibited *Col1a1* mRNA levels to various degrees in the absence of osteogenic inducers. These results may be associated with the increase of *Runx2* mRNA and protein expression triggered by FGF-2 and are similar to previously reported studies, which identified that the addition of FGF-2 to culture media suppressed the expression of *Col1a1* mRNA at all time-points <14 days (29,34,35). Therefore, in the present culture system, FGF-2 induced Runx2 protein expression but suppressed the mineralization of hPDLCs by affecting *Col1a1* mRNA expression. Additionally, DEX has been demonstrated to inhibit the expression of *Col1a1* mRNA in immortalized hMSCs during osteogenic differentiation (36). This function-suppressing differentiation of hPDLCs into mineralized tissue may aid in the stabilization of the fibroblastic phenotype and maintenance of hPDL structural integrity.

EGFR is highly upregulated in periodontal disease and may have a pivotal role in regulating cell migration, proliferation and epithelial wound healing (37,38). A previous study has reported that the EGF/EGFR system regulates the phenotype of different cell populations (39), and pre-osteoblasts and prechondrocytes have high levels of EGFR *in vivo*. However, the amount diminishes significantly as differentiation progresses. Mature osteoblasts and chondrocytes do not express EGFR (40,41), and in addition, EGFR on hPDLCs functions as a negative regulator of their differentiation into mineralized tissue-forming cells (12). In the present study, the gene expression of *EGFR* was significantly upregulated by FGF-2 and osteogenic inducers compared with hPDLCs cultured in the GM, but downregulated by FGF-2 in the presence of osteogenic inducers. Thus, we hypothesize that the upregulation of the *EGFR* gene may have an important role in maintaining the hPDLC phenotype, balancing the population of fibroblasts in the hPDL by inhibiting their differentiation into mineralized tissue-forming cells.

In conclusion, the results of the present study demonstrate that FGF-2 facilitates hPDLC proliferation in the absence and presence of osteogenic inducers, while modulating the balance between their osteoblastic and fibroblastic phenotypes by affecting the gene expression of *Runx2*, OCN, *Col1a1* and *EGFR* in the absence of ostoegenic inducers, in addition to inhibiting the mineralization of hPDLCs in the presence of osteogenic inducers. These results suggested that FGF-2 induces the growth of immature hPDLCs, which is a competitive inhibitor of epithelial downgrowth, and suppresses their differentiation into mineralized tissue by affecting Runx2 expression. This may lead to the acceleration of periodontal regeneration.

## Figures and Tables

**Table I:** Primer sequences used for RT-qPCR.

Genes	Primers	Size, bp
*GAPDH*	F: CATGTTCCAATATGATTCCACC R: GATGGGATTTCCATTGATGAC	88
*Runx2*	F: CCAACCCACGAATGCACTATC R: TAGTGAGTGGTGGCGGACATAC	91
*Col1a1*	F: GAACGCGTGTCATCCCTTGT R: GAACGAGGTAGTCTTTCAGCAACA	91
OCN	F: CCTGAAAGCCGATGTGGT R: GGCAGCGAGGTAGTGAAGA	148
*EGFR*	F: GGAGAACTGCCAGAAACTGACC R: GCCTGCAGCACACTGGTTG	106

RT-qPCR, reverse transcription-quantitative polymerase chain reaction; bp, base pairs; *Col1a1*, collagen type I, α1; OCN, osteocalcin; *EGFR*, epidermal growth factor receptor; F, forward; R, reverse.
